# Community Mortality Due to Respiratory Syncytial Virus in Argentina: Population-based Surveillance Study

**DOI:** 10.1093/cid/ciab497

**Published:** 2021-09-02

**Authors:** Mauricio T Caballero, Alejandra M Bianchi, Sebastian Diaz Grigaites, Paola X De la Iglesia Niveyro, Alejandra Nuño, Sandra Valle, Gabriela Afarian, Sebastian A Esperante, Adrian J P Ferretti, Sofia Jares Baglivo, Julian De Luca, Damian Alvarez-Paggi, Adriana Diamanti, Quique Bassat, Fernando P Polack, RSV mortality network, Cristian M Zea, Paula Caporal, Natalia Rakislova, Rosauro Varo, Juan Carlos Hurtado, Marcelo Isaac Dabbah, Ana María Carballo, Lorena Madrid, Patricia Ares, Gabriela Hernandez, Lucas Basanta

**Affiliations:** 1 Fundacion INFANT, Buenos Aires, Argentina; 2 Consejo Nacional de Investigaciones Científicas y Técnicas (CONICET), Buenos Aires, Argentina; 3 Morgue Judicial del Instituto de Ciencias Forenses Conurbano Sur, Ministerio Publico de la Provincia de Buenos Aires, Lomas de Zamora, Argentina; 4 Hospital Italiano de Buenos Aires, Servicio de Anatomia Patologica, Buenos Aires, Argentina; 5 ISGlobal, Hospital Clínic-Universitat de Barcelona, Barcelona, Spain; 6 Centro de Investigação em Saúde de Manhiça (CISM), CP Maputo, Mozambique; 7 ICREA, Pg. Lluís Companys, Barcelona, Spain; 8 Pediatric Infectious Diseases Unit, Pediatrics Department, Hospital Sant Joan de Déu (University of Barcelona), Barcelona, Spain; 9 Consorcio de Investigación Biomédica en Red de Epidemiología y Salud Pública (CIBERESP), Madrid, Spain

**Keywords:** acute respiratory infections, Infant community mortality, minimal invasive tissue sampling, respiratory syncytial virus

## Abstract

**Background:**

Many deaths in infants from low-middle income countries (LMICs) occur at home or upon arrival to health facilities. Although acute lower respiratory tract illness plays an important role in community mortality, the accuracy of mortality rates due to respiratory syncytial virus (RSV) remains unknown.

**Methods:**

An active surveillance study among children aged under 5 years old (U5) was performed in Buenos Aires, Argentina, between January and December 2019, to define the burden and role of RSV in childhood community mortality.

**Results:**

A total of 63 families of children U5 participated in the study. Based on a combined approach of tissue sampling, verbal autopsies, and expert’s analysis, RSV infection was found in the causal chain of 11 from 12 cases with positive molecular biology results in respiratory samples. The estimated mortality rate due to RSV among infants was 0.27 deaths/1000 live births. The mean age of RSV-related household deaths was 2.8 months of age (standard deviation [SD] 1.7), and 8/12 were male infants (66.7%). Dying at home from RSV was associated with *Streptococcus pneumoniae* and/or *Moraxella catarrhalis* lung coinfection (75%), living in slums and settlement (odds ratio [OR], 17.09; 95% confidence interval [CI], 1.3–219.2), and other underlying comorbidities (OR, 14.87; 95% CI, 1.3–164.6).

**Conclusions:**

Infant community mortality rates due to RSV are higher than those reported in industrialized countries and similar to those reported in hospital-based studies in the same catchment population.

Respiratory syncytial virus (RSV) is a major cause of acute lower respiratory tract illness (LRTI) in infants worldwide. The contribution of this pathogen to child mortality has been underestimated for many reasons [1–3]. Some of those are well known, such as lack of active RSV surveillance programs, a paucity of molecular diagnosis, a low rate of complete diagnostic autopsy, and a high contribution of community mortality in poor resource settings [1, 2, 4]. Other causes are less understood such as the uncertainty related to RSV death attribution based on nonrespiratory deaths or bacterial coinfection can tip the scale and bias the RSV burden estimation [5–7]. All of these scenarios are commonplace in resource-constrained settings where 99% of the RSV-related deaths occur [1]. In this context preemptive interventions are challenging because decision makers are hampered by the scarcity of information on mortality due to RSV, mainly at the community level [8, 9]. Therefore, until vaccine trials can provide a more accurate understanding, it is crucial to design studies that allow us to acknowledge not only the burden of mortality but also to assess possible pathobiological mechanisms of RSV related fatalities.

Our group pioneered building a population-based community mortality platform in a resource-constrained area of Buenos Aires, where we reported for the first time that RSV was found in the nasopharynx of some babies who died at home, in circumstances of high structural poverty [8]. Home mortality rates for infants due to RSV were high (0.26/1000 live births) and similar to those reported for hospitalized children (0.28/1000 live births) [8, 10, 11].

In 2019, we expanded our program to a combined approach based on active surveillance, minimal invasive tissue sampling technique (MITS), verbal autopsy (VA), molecular methods, and cause of death (COD) attribution based on the determination of Cause of Death (DeCoDe) process from the CHAMPS platform [5, 6, 8, 9, 12]. This study aimed to accurately define the role of RSV in the causal chain of community deaths in children under 5 years old (U5).

## METHODS

### Study Population

We designed an active surveillance population-based prospective study, in collaboration with a judicial morgue where six districts of the Province of Buenos Aires sent U5 community death children for necropsy. The study was conducted between 1 January and 31 December 2019, in a catchment population of 40 027 live births/year [8]. Inclusion criteria were: (a) all children U5 who died at home (outside any health facility), (b) when certified as “dead upon arrival” by a physician at a hospital, and (c) when classified as unusual, suspicious, or unknown cause and full necropsy was requested by a local prosecutor. Dead children were excluded if there was a criminal or external underlying cause. To compare and explore for risk factors in home deaths due to RSV, healthy controls from the same catchment area were enrolled by social workers.

### Study Procedures

Study coordinators screened for eligible U5 deaths within the defined populations and approached families for informed consent procedures and grief counseling. In those subjects included in the study, a MITS procedure was conducted within 24–48 hours of death, samples were obtained from both lungs, liver, heart, brain, blood, rectal and nasopharyngeal swabs, and were frozen at −80°C. Additional tissue samples were fixed for 6–48 hours in formaldehyde 10% buffer. Between 30 and 90 days after death a team of a social worker and a pediatrician interviewed the parents or direct relatives to obtain clinical and epidemiological data (VAverbal autopsy), following the standard 2016 VA tool [8]. All the samples were processed by simplex quantitative polymerase chain reaction (qPCR) for 16 pathogens diagnosis, and by hematoxylin-eosin (HE), and immunohistochemistry (IHC) for RSV [8, 10, 11]. After all the procedures were performed, a cause of death was attributed by an expert panel using DeCoDe standards (Figure S1) [5, 13]. For control subjects enrollment, a team of social workers visited families randomly with U5 children from the same capture area and interviewed them. All the data were stored in REDCap (Figure S1). The State’s Institutional Review Board approved the study.

### RSV Cause of Death Attribution Criteria

The attribution of the COD was conducted using the standard DeCoDe procedure through 2 international independent expert panels (composed by pediatricians, neonatologists, epidemiologists, pathologists, and microbiologists) who reviewed each case using all VA data, molecular biology results, histopathology findings, and necropsy reports (where relevant medical data are collected from primary sources) and seek consensus to determine the most likely causes and chain of events leading to death [5, 12]. The coding of each COD was assigned using a mortality classification system derived from the International Statistical Classification of Disease and Related Health Problems, 11th revision from World Health Organization (WHO) (ICD-11), and for perinatal cases a specific version was used (ICD-PM) [5, 13]. The DeCoDe process included documenting the entire causal chain leading to death, as in a WHO standard death certificate, and categorize a certainty level of diagnosis for each step [6]. In order to be included in the causal chain, it was considered whether appropriate management or prevention of that condition could have prevented death [6].

### Nucleic Acid Purification

All tissue samples for microbiological analyses were stored at − 80°C until the DNA/RNA purification. Tissue cores obtained by the MITS procedures were lysated at 55°C using a dry block heater (Thermo Scientific™) in 200 µL of ATL lysis buffer (Qiagen), 20 µL of 10 mg/mL proteinase K (Qiagen), and 5 µL β-mercaptoethanol (Sigma-Aldrich). Tissue samples were homogenized by vortexing during lysis. Nucleic acid extractions (DNA + RNA) were performed using a Pathogen’s DNA/RNA purification kit (Applied Biosystems™) in a semi-automated system (MagMAX™ Express-96 Deep Well Magnetic Particle Processor) [9, 14]. All samples were analyzed for nucleic acid concentration (ng/µL) and purity (260/280 absorbance ratio) using a full spectrum (220–750 nm) spectrophotometer (Thermo Scientific™).

### Microbiological and Histological Analysis

To investigate etiologic agents of community deaths, we processed all samples by qPCR and IHC. In all subjects, exploration for incident respiratory pediatric pathogens was conducted as previously described [8–11, 14]. Tissue samples were subjected to routine processing procedures such as formalin fixation, paraffin embedding, slide sectioning, and H&E staining. Immunohistochemistry assay for RSV was performed on an automatized Benchmark staining module (Tucson, Arizona) using clone RSV5A6(Abcam). Samples were considered positive for RSV if moderate to strong cytoplasmic stain was observed in macrophages and/or respiratory epithelium. Lung sections were assessed and scored in 4 categories based on the severity by a trained pathologist using a previously described criteria [15].

### Statistical Analysis

Population’s live births information was derived from the state regional surveillance system in the catchment area to estimate community mortality rates due to RSV [16]. Frequency distribution, mean, median, interquartile range (IQR), standard deviation (SD), and 95% confidence interval (95% CI) were used for variables description as appropriate. For the univariate comparative analysis between deaths due to RSV disease versus healthy controls, we used Fisher exact test for dichotomic variables (based on the small sample size in the RSV death group), rank sum Mann-Whitney test for nonparametric, and *t* test for parametric continuous variables. Those variables with *P* ≤ .10 in univariable models were considered for inclusion in a hierarchical multivariable logistical regression analysis, considering as outcome mortality due to RSV (yes/no) [8, 10, 17–19]. Cohen’s kappa coefficient (κ) was used to measure DeCoDe inter-rater reliability between expert panels, agreement scale was categorized as slight (<0.2), fair (0.21–0.40), moderate (0.41–0.60), substantial (0.61–0.80), and almost perfect (>0.80). A *P* <.05 was considered to indicate statistical significance. Statistical analyses were performed using Stata version 15 (StataCorp).

## RESULTS

### Burden of Community Mortality Due to RSV

Our program screened a susceptible population living in 6 counties in the southern peripheries of Buenos Aires, Argentina. We included 63 U5 children who died at home or “upon arrival” at any health facility of the region from 68 eligible subjects (92.7% of parents’ acceptance). We were able to conduct VA interviews in 90% of the parents/guardians of the participant subjects. Verbal autopsy refusal causes were bereaved parents, who did not want even grief counseling interviews and moved out the catchment area after child death. Nevertheless, in all cases necropsy information was obtained to complement relevant demographic and medical records. We obtained 100% of tissue samples for histopathology and qPCR analysis by MITS. The population in this area without medical insurance (84% of the families enrolled) receives free health care from a network of primary care centers (PCC) and 7 pediatric departments in general hospitals.

**Figure 1.**
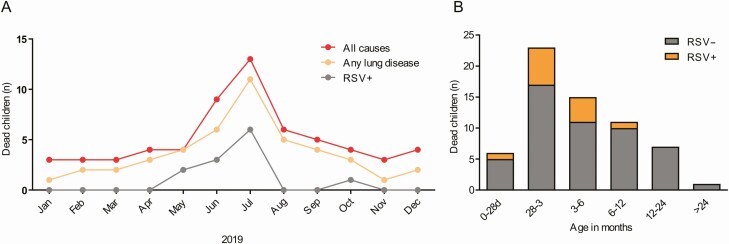
Burden of community mortality due to RSV in infants. *A*, Frequency of children deaths in the community by month of chronologic age. *B*, Infant deaths at community by month in 2019. All-cause of deaths (*red line*), all respiratory causes of death (*orange line*), RSV deaths (*gray line*). Abbreviation: RSV, respiratory syncytial virus.

RSV was confirmed in 12 of 63 community deaths (19.1%) in the catchment region, mean age was 2.8 months (SD 1.7, IQR 1.7–3.6), all cases were < 6 months of age (1 neonate), and 8/12 were male (66.7%). RSV related deaths occurred mostly during cold seasons 11/32 (34.4%), except for one death (Figure 1A, 1B). From all tissue samples analyzed for RSV, 6 NP swabs (50%), 11 lung cores (91.7%), and 2 blood/liver samples (16.7%) were positive by qPCR, while 8 lung slides (66.7%) were positive by IHC. After DeCoDe expert panels reviewed all cases, RSV was found in 11 cases in the chain of events leading to death (83% inter-rate agreement, kappa 0.43, *P* = .035). The *infant community mortality rate* due to RSV was 0.27 per 1000 live births during the study period, very similar to our previous findings [8, 10, 11]. All RSV-related deaths were from A strain, just one case was infected with both strains together. Furthermore, we found a subject with a previously microbiologically confirmed RSV pneumonia during a long hospitalization two months before the RSV-related demise.

The sensitivity and specificity of the nasopharyngeal swab for RSV by qPCR was 45.45% and 98.08%, respectively (ROC 0.72, 95% CI .56–.87), whereas negative and positive predictive value was 83.33% and 89.47%, respectively. Although RSV positive by IHC had a sensitivity of 72.73% and a specificity of 100% (ROC 0.86, 95% CI .73–1), lung RSV confirmed by qPCR had a sensitivity and specificity of 100%.

### Histopathological Findings in RSV Related Demises

**Figure 2.**
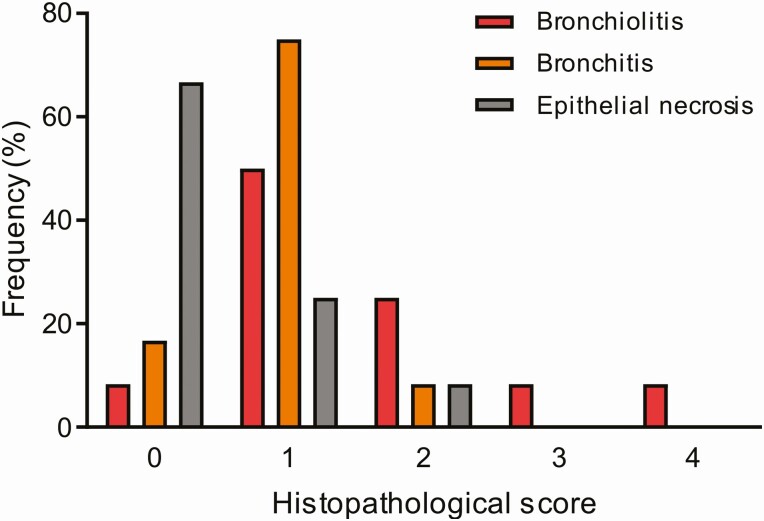
Histopathology lung severity score in infants who die due to RSV. Frequency of different pattern of lung histopathology was measured in 4 categories based on bronchiolitis (*red bar*), bronchitis (*orange bar*), and epithelial necrosis (*gray bar*) severity. Bronchiolitis score was: 0 = no remarkable lesions, 1 = minimal detectable epithelial degeneration in few bronchioles, 2 = epithelial degeneration in <10% of the airway lumen, 3 = epithelial degeneration in >10–50% of the lumen with cell debris and neutrophils, 4 = circumferential bronchiolitis with dense adventitial lymphocytes in multiple bronchioles [15]. Abbreviation: RSV, respiratory syncytial virus.

**Figure 3.**
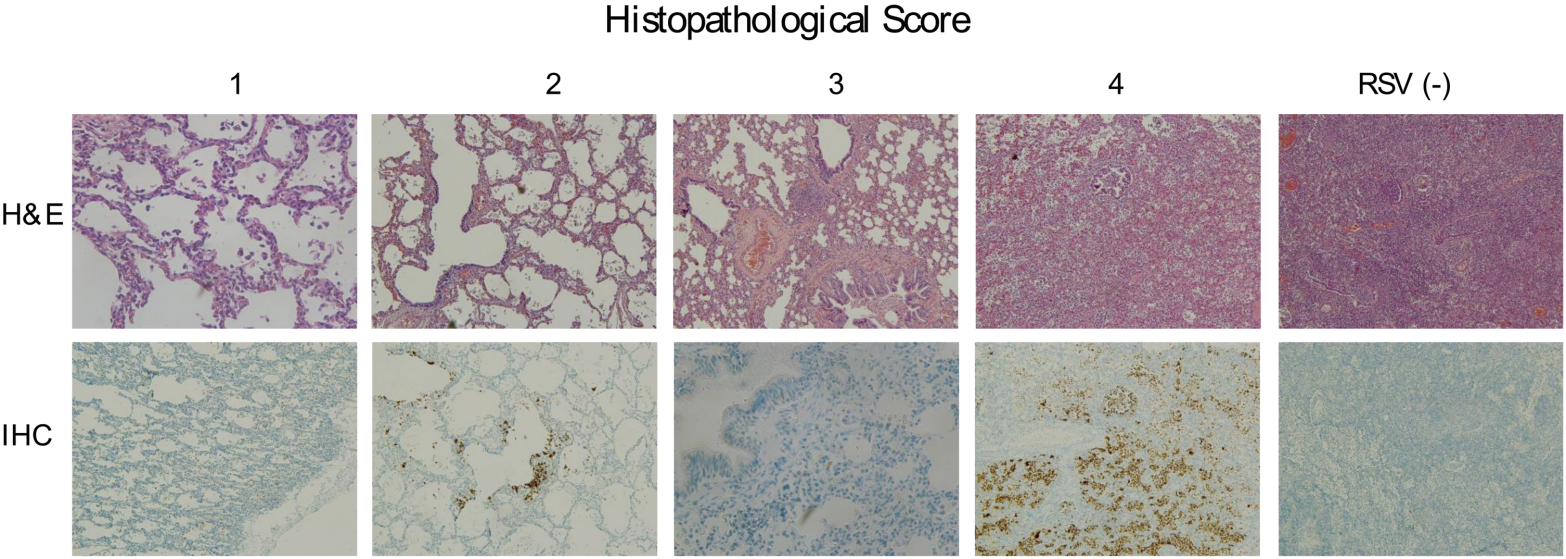
Microscopic lung lesions in infants with bronchiolitis due to RSV. Lung samples were stained with hematoxylin and eosin and with immunohistochemistry for RSV. Scores were indicated from 1 to 4 as previously described. Additionally, there was a control lung tissue of an infant who died at hospital from pneumonia without RSV infection. Abbreviations: IHC, immunohistochemistry; RSV, respiratory syncytial virus.

Even though RSV was detected in respiratory and systemic samples by molecular biology methods and that 2 independent experts determined the presence of the virus in the causal chain, we could not explain the mechanism underlying RSV mortality in most of the children of the study. Our hypothesis encompassing lung histopathology due to RSV drove us to expect a severe lung consolidation in RSV deaths, with clear signs of respiratory distress, like those found in children admitted in critical care services [6, 10]. However, we found in 8/12 (66.7%) deaths a mixed histopathology pattern of mild bronchiolitis and interstitial pneumonia (Table 1), and no RSV case had the characteristic syncytia in lung parenchyma [20, 21]. From all the RSV deaths we noticed that just 16.7% had severe acute lung disease by histopathology (Figures 2–3). Indeed, most of the cases had mild histopathological outcomes (scoring 0–2) that makes us doubt about the underlying mechanism of disease. Immunohistochemistry staining was a trustworthy technique to demonstrate RSV percentage of lung parenchyma infection (Figure 3), and the viral pathophysiological role.

**Table 1.** Characteristics of Infants Who Died Due to RSV Infection

						RSV						
No.	Age (m)	Sex	Underlying Disease	Illness Duration	Lung Histopathology	NP	Lung	IHC	Coinfection	COD	Causal chain	Certainty level
1	3.2	Male	Undernutrition	Sudden	Bronchiolitis/pneumonia	Pos	Pos	Pos	SP/MC	CA41.0 acute bronchiolitis due to RSV	Yes	1
2	3.5	Male	None	Sudden	Bronchiolitis	Neg	Pos	Neg	None	CA41.0 acute bronchiolitis due to RSV	Yes	1
3	3.7	Male	None	Sudden	Bronchiolitis/pneumonia	Neg	Pos	Pos	MC/SA	CA41.0 acute bronchiolitis due to RSV	Yes	1
4	2.1	Male	Genetic Syndrome	Sudden	Bronchiolitis/pneumonia	Pos	Pos	Pos	SP	CA41.0 acute bronchiolitis due to RSV	Yes	1
5	1.5	Male	Undernutrition	Sudden	Bronchiolitis/pneumonia	Neg	Pos	Pos	None	CA41.0 acute bronchiolitis due to RSV	Yes	1
6	1.9	Female	Undernutrition	4 days	Bronchiolitis/pneumonia	Pos	Pos	Pos	SP/MC	CA40.07 Pneumonia due to *Streptococcus* sp.	Yes	1
7	6	Female	Trisomy 18	4 days	Bronchiolitis/pneumonia	Pos	Pos	Pos	MC	CA41.0 acute bronchiolitis due to RSV	Yes	1
8	5.9	Female	None	16 days	Bronchiolitis/pneumonia	Neg	Pos	Pos	None	CA41.0 acute bronchiolitis due to RSV	Yes	1
9	1.3	Male	None	2 days	Bronchiolitis	Neg	Pos	Pos	SP/PIV3	CA41.0 acute bronchiolitis due to RSV	Yes	1
10	1.9	Female	Undernutrition	2 days	Bronchiolitis/pneumonia	Pos	Pos	Neg	SP	CA40.07 pneumonia due to *Streptococcus* sp.	Yes	1
11	0.9	Male	Undernutrition	Sudden	Pneumonia	Pos	Neg	Neg	SP/MC	CA40.07 pneumonia due to *Streptococcus* sp.	No	3
12	1.9	Male	None	Sudden	Vascular congestion	Neg	Pos	Neg	None	CA41.0 acute bronchiolitis due to RSV	Yes	3

Abbreviations: COD, cause of death; IHC, immunohistochemistry; MC, *Moraxella catarrhalis*; NP, nasopharyngeal swab; PIV3, parainfluenzavirus3; RSV, respiratory syncytial virus; SP, *Streptococcus pneumoniae*.

Bacterial coinfection was a common finding in our series (66%), but contrarily to our presumptions, RSV drove lung parenchyma compromise based on the percentage and characteristics of the main histopathology pattern we found in many cases. Very few tissues had clear bronchopneumonia histopathological diagnosis. We found that *Streptococcus pneumoniae* (50%) and *Moraxella catarrhalis* (41.6%) coinfect lung tissues of infants with RSV LRTI and even cause pneumonia. Additionally, we observed that 9/12 (75%) of these subjects had a nasopharyngeal bacterial carriage, and adding all results we detected that all 12 subjects with RSV infection had at least one additional sample coinfected with some bacteria.

### Housing Conditions and Family Habits in RSV Home Mortality

We investigated whether community mortality due to RSV was influenced by environmental and social factors that preceded birth, by comparing housing and social conditions with 171 healthy controls from the same catchment area (Table 2). Similar to our previous studies, families of infants who die due to RSV live more often in slums and settlements (OR 42.2, 95% CI 2.4–730.4, *P* = .010), in overcrowded houses, with scarce access to public transport than healthy controls (Table 2). Mothers with incomplete education, insufficient social networks, inadequate prenatal care, and incomplete vaccination during pregnancy have been a common characteristic in the region. Even 58.3% of the mothers and families had a stressful situation before the death event in the RSV group (violence situations, power outage, and relatives with a severe disease). Additionally, we detected that infants from mothers who delivered by cesarean procedure were more often associated with RSV mortality in our population (OR 6.7, 95% CI 1.2–37.5, *P* = .030).

**Table 2.** Univariable Analysis

	RSV Deaths	Healthy Control	*P*
	(n = 12)	(n = 171)	
	n/N (%)	n/N (%)	
Housing and neighborhood conditions			
Slum and settlement	7/12 (58.3)	22/168 (13.1)	.001
Running water	10/12 (83.3)	164/168 (97.6)	.792
Mud street	8/12 (66.7)	92/171 (53.8)	.58
Lack of public transport	5/12 (41.7)	26/171 (15.2)	.06
Overcrowding (>3 person per bedroom)	7/12 (58.3)	54/171 (31.6)	.060
Family habits and socioeconomic support			
Tobacco smoking at home	6/11 (54.6)	62/170 (36.5)	.189
Incomplete maternal education	10/12 (83.3)	127/171 (74.2)	.751
Lack or inadequate prenatal care	3/12 (25.0)	41/171 (23.9)	.199
Mother with poor social network	3/11 (27.3)	23/168 (13.7)	.202
Stressful situation (previous week)	7/12 (58.3)	42/171 (24.6)	.017
Caesarean delivery	7/11 (58.3)	44/169 (25.8)	.013
Biological characteristics			
Male sex	8/12 (66.7)	80/170 (47.1)	.155
Prematurity	2/12 (16.7)	19/171 (11.1)	.411
NICU admission	4/12 (33.3)	31/171(18.1)	.127
Low birth weight	2/12 (16.7)	11/171(6.4)	.205
Perinatal infection	1/11 (9.1)	2/162 (1.23)	.165
Underlying disease	7/12 (58.3)	28/171 (16.4)	.002
Care behaviors			
No breastfeeding in < 12 months	3/11 (27.3)	9/100 (9)	.097
Incomplete vaccination	2/11 (18.2)	18/169 (10.5)	.351
Risk for SIDS	8/12 (66.7)	107/171 (62.6)	.181
Inadequate health controls	3/11 (27.3)	63/171 (36.8)	.386
Signs of moderate or severe illness by VA	2/12 (16.7)	62/171 (36.3)	.143
PCC/ED visit during last illness	9/11 (81.8)	80/82 (97.6)	.067

Abbreviations: ED, emergency department; NICU, neonatal intensive care unit;

PCC, primary care center; RSV, respiratory syncytial virus; SIDS, sudden infant death syndrome; VA, verbal autopsy.

### Underlying Diseases in RSV Mortality

Next, we investigated whether infants who died from RSV had differential biological background against healthy controls. Comparisons among both groups revealed similar frequency of prematurity, and low birth weight. Fatalities related to RSV illness are usually associated with the presence of comorbidities [10, 22]. Thus, we explored this in the study, and we found that 58.3% of infants who died due to this virus had an underlying disease (OR 14.8, 95% CI 1.3–164.6, *P* = .038) in comparison with healthy controls (Table 3). The most frequent comorbidity was undernutrition (41%), being “young and light” influenced poor RSV outcomes, in our population as we had previously shown [10, 11, 17].

**Table 3.** Hierarchical Multivariable Analysis: Odds Ratio for Death Due to RSV Illness

	Level 1		Level 2		Level 3	
	Odds Ratio (95% CI)	*P*	Odds Ratio (95% CI)	*P*	Odds Ratio (95% CI)	*P*
Slum and settlement	7.67 (1.8–32.7)	.006	7.37 (1.2–43.8)	.028	17.09 (1.3–219.2)	.029
Lack of public transport	1.03 (.3–3.9)	.964	1.05 (.1–7.6)	.964	1.13 (.1–15.8)	.927
Overcrowding(*>*3 person per bedroom)	1.88 (.52–6.8)	.334	4.04 (.7–23.1)	.115	2.20 (.2–21.3)	.505
Stressful situation(previous week)			4.06 (.83–19.8)	.083	5.18 (.5–50.3)	.156
Caesarean delivery			6.71 (1.2–37.5)	.030	8.36 (.7–98.9)	.092
Underlying disease			8.45 (1.6–45.4)	.013	14.87 (1.3–164.6)	.028
No breastfeeding in <12 months					0.95 (.1–10.1)	.967
PCC/ED visit during last illness					0.01 (.01–12.9)	.524

Abbreviations: CI, confidence interval; ED, emergency department; NICU, neonatal intensive care unit; PCC, primary care center; RSV, respiratory syncytial virus.

### RSV-related Deaths in Those Cases that were Attributed to Sudden Infant Death Syndrome

Finally, we explored how families had taken care of their babies after birth. Lack of breastfeeding and incomplete vaccination were slightly more frequent in the RSV group, but without statistical significance. To understand RSV mortality prevention strategies in poor settings in greater depth, it is extremely relevant to determine parental awareness for severe signs, and to assess how early do parents visit a health facility when they recognized an illness. Thus, we noticed that some parents recognized severe signs of illness (16%), and most of them visited a health facility (81%) during the days preceding the death (Table 2). Indeed, families whose baby had a doctor’s diagnosis of bronchiolitis attended several times for medical care, where health workers missed valuable opportunities to prevent those babies’ fatal outcomes. Thus, mortality due to RSV could not be prevented despite the constant visits of families to health centers.

When we explored infants’ sleep behaviors, we found that in general parents co-slept with them (>60%), but as we had previously shown, this was not associated with home deaths. In 3 RSV cases forensic staff found signs of milk aspiration that were correlated with parents’ description of the fatal event. In one case we found a medical record of apneas certified by a neonatologist and treated with caffeine during neonatal intensive care unit (NICU) hospitalization, which was a key finding for the attribution of the immediate COD. Actually, we detected that 66.7% of the RSV-related demises were attributed to sudden infant death syndrome (SIDS) as a COD after necropsies. Even so, many deaths were unexpected based on families’ reports (“the baby went to sleep and never woke up”). By this multidisciplinary approach we were able to determine that SIDS were overestimated and that RSV was associated with those deaths as previously described [16, 20, 23–25].

## Discussion

In this study, we found that RSV community mortality rate for 2019 equals hospital mortality rates previously showed in the same catchment area [8, 10, 11]. All RSV cases reported were in infants equal or under the age of 6 months. Underlying illnesses, parental reported sudden fatal event, and bacterial coinfection were also found in more than half of the cases. In fact, RSV kills infants in 2 different sceneries: one at medical facilities, often experiencing pneumothorax and/or bacterial sepsis; and a second group dying in the community in association with social inequalities, a mixed pattern of mild, bacterial coinfection, and illness underestimation by parents and physicians [6, 8, 10, 11, 17]. We also considered that physicians/parents’ disease underestimation was aligned with the paucity of clinical signs presented in many cases. In a sense, RSV mortality in low-resource settings underlines the profound structural problems in public health services and in sociodemographic determinants. Furthermore, home mortality due to RSV can also underlay a phenotype of mild disease that forensic physicians could misdiagnose with sudden death syndrome during death certification [1, 4, 8, 10, 26, 27].

We found that RSV can directly kill per se, but often requires a synergistic co-factor to kill [8, 10, 17, 22, 28]. *Streptococcus pneumoniae* and *Moraxella catarrhalis* were found in 66% of lungs with histopathological signs of pneumonia from RSV-related deaths in this study. Indeed, it is well known that RSV and other respiratory viruses causes functional changes in respiratory epithelial cells facilitating adherence and invasion of bacteria [29–32]. Undernutrition was the main premorbid condition associated to RSV mortality in this study, contrasting with industrialized countries where RSV deaths are infrequent and commonly associated with more complex chronic conditions [22, 33].

Numerous home deaths in our study occurred during sleep, in a silent way, with mild bronchiolitis or even without any apparent lung disease; perhaps infants died with RSV by hypoxemia, caused by milk aspiration, or apnea [20, 23, 24]. Apnea is a tricky event, hard to diagnosis, not well understood, and when appears in young babies, quick actions must be taken [34, 35]. Interestingly RSV is a common cause of apnea, in an earlier study we found that this virus caused 32% of apneic syndrome detected in hospitalized infants [17]. Therefore, a deep understanding of RSV-related apnea can be important to plan preemptive strategies in the future.

This study presents several caveats. First, findings may vary in scope and magnitude in other countries. Indeed, it would be key to elucidate through well designed studies in different regions the burden of mortality due to RSV by age group to determine if necessary, a mortality surveillance in children older than 6 or 12 months is needed. Second, the detection of bacterial pathogens was performed only by RT-PCR assays (no specific immunohistochemistry was performed), which hamper the precision of the understanding of bacterial findings in the chain of events leading to death. Third, VA data recording may have parental remember bias.

By a multidisciplinary approach we were able to determine with a high level of certainty that RSV was in the causal chain of home deaths in Argentina, and this outstrip those reported in industrialized countries [22, 26, 33]. Families of poor resources are at higher risk of suffering fatal outcomes when their infants are infected with respiratory pathogens. Access to healthcare and empowering medical services to bring adequate treatment of young infants with RSV LRTI needs are an urgent consideration to achieve reductions in childhood deaths [4, 8, 10, 28]. Finally, we showed that RSV bronchiolitis could be a consequence of different pathophysiological basis of disease. Therefore, we must grasp the nettle and shrewdly use the resources to discriminate these RSV associated endotypes by transcriptomic approach, machine learning analysis, and personalized based therapeutics [36, 37].

## Supplementary Data

Supplementary materials are available at *Clinical Infectious Diseases* online. Consisting of data provided by the authors to benefit the reader, the posted materials are not copyedited and are the sole responsibility of the authors, so questions or comments should be addressed to the corresponding author.

## Notes

***Acknowledgments.*** We want to thank to the Ministerio Publico de la Provincia de Buenos Aires, specially to the General Attorney Dr Julio Conte-Grand, and to Dr Francisco Pont Verges, and Dr Alejandra Attarian Mena. We also want to thank the Lomas de Zamora Judicial Morgue staff and its director Dr Hernan Junod, and especially to all of the beavered families who selflessly trusted in this study.

RSV mortality network* members are

Cristian M Zea MD10, Paula Caporal MD11, Natalia Rakislova MD PhD5, Rosauro Varo MD5, Juan Carlos Hurtado MD5, Marcelo Isaac Dabbah MD3, Ana María Carballo MD3, Lorena Madrid MD3, Patricia Ares MD3, Gabriela Hernandez MD3, Lucas Basanta BSc3

1Fundacion INFANT, Argentina; 2Consejo Nacional de Investigaciones Científicas y Técnicas (CONICET), Buenos Aires, Argentina; 3Morgue Judicial del Instituto de Ciencias Forenses Conurbano Sur, Ministerio Publico de la Provincia de Buenos Aires, Argentina, 4Hospital Italiano de Buenos Aires, Servicio de Anatomia Patologica, Buenos Aires, Argentina. 5ISGlobal, Hospital Clínic—Universitat de Barcelona, Barcelona, Spain. 6Centro de Investigação em Saúde de Manhiça (CISM), CP Maputo, Mozambique. 7ICREA, Pg. Lluís Companys, Barcelona, Spain. 8Pediatric Infectious Diseases Unit, Pediatrics Department, Hospital Sant Joan de Déu (University of Barcelona), Barcelona, Spain. 9Consorcio de Investigación Biomédica en Red de Epidemiología y Salud Pública (CIBERESP), Madrid, Spain. 10Hospital Nacional de pediatría “Prof. Dr Juan P. Garrahan,” Buenos Aires, Argentina, 11Hospital Interzonal de Agudos Especializado en Pediatría “Sor María Ludovica,” La Plata, Argentina.

***Supplement sponsorship.*** This supplement is sponsored by the Bill and Melinda Gates Foundation.

***Financial support.*** This publication is based on research funded by the Bill & Melinda Gates Foundation to F. P. P.

***Potential conflict of interest.*** F. P. P. reports grants and personal fees (Advisory Board Agreement) from Janssen, grants and personal fees (Consulting Agreement) from Novavax, Inc, personal fees (Consultancy Agreement) from Bavarian Nordic A/S, personal fees (Advisory Board Agreement) from Pfizer, personal fees (Advisory Board Agreement) from Sanofi, personal fees (Consultant Agreement) from Regeneron, personal fees (Consultant Agreement) from Merck, outside the submitted work. Q. B. reports being a Member of the Independent Data Monitoring Committee (IDMC) for GlaxoSmithKline (GSK) for respiratory syncytial virus (RSV) vaccine development for the protection of Infants (since November 2015). All other authors report no potential conflicts. All authors have submitted the ICMJE Form for Disclosure of Potential Conflicts of Interest. Conflicts that the editors consider relevant to the content of the manuscript have been disclosed.
